# Corticosteroid dose increase is a risk factor for nonalcoholic fatty liver disease and contralateral osteonecrosis of the femoral head: a case report

**DOI:** 10.1186/s12891-019-2468-5

**Published:** 2019-02-19

**Authors:** Hirokazu Shimizu, Tomohiro Shimizu, Daisuke Takahashi, Tsuyoshi Asano, Ryuta Arai, Yasunari Takakuwa, Norimasa Iwasaki

**Affiliations:** 1 Department of Orthopaedic Surgery, Faculty of Medicine and Graduate School of Medicine, Hokkaido University, Kita-15 Nishi-7, Kita-ku, Sapporo, 060-8638 Japan; 2 Department of Pathology, Sapporo Medical Center NTT EC, Nippon Telegraph and Telephone East Corporation, Sapporo, Japan

**Keywords:** Osteonecrosis of the femoral head, Corticosteroid, Nonalcoholic fatty liver disease

## Abstract

**Background:**

The incidence of bilateral corticosteroid-induced osteonecrosis of the femoral head (ONFH) is high. Although the precise mechanism of corticosteroid-induced ONFH development is unclear, hepatic enzyme abnormalities such as low activity of hepatic cytochrome P450 3A could be one cause. Herein, we report the case of a patient who developed ONFH in the contralateral hip after the dose of corticosteroids for idiopathic thrombocytopenic purpura was increased. Liver biopsy was done to rule out autoimmune hepatitis.

**Case presentation:**

A 32-year-old woman had been treated with continuous corticosteroids of up to 10 mg/day for Sjögren’s syndrome for 25 years and corticosteroid-induced ONFH in the left side. At age 33, idiopathic thrombocytopenia developed, which was treated by increasing the corticosteroid dose (40 mg/day). Two months later, liver enzyme level began to increase slightly and continued to increase. A year after corticosteroid dose increase, contralateral ONFH developed, and a liver biopsy demonstrated nonalcoholic fatty liver disease (NAFLD).

**Conclusions:**

The current case indicates that corticosteroid dose increase is a potential risk factor for NAFLD and contralateral ONFH. Therefore, it would be useful and important for to screen and monitor patients with hepatic enzyme abnormality for ONFH occurrence.

## Background

Non-traumatic osteonecrosis of the femoral head (ONFH) is believed to be a multifactorial disease, with high-dose corticosteroid therapy for inflammatory diseases and alcohol abuse considered major risk factors [1, 2]. ONFH can be bilateral in up to 60% of cases at initial diagnosis [3]. In contrast, patients with unilateral ONFH rarely develop a subsequent osteonecrotic lesion in the contralateral femoral head. To our knowledge, there have been three cases of new corticosteroid-induced ONFH developing in the contralateral femoral head [4–6].

The underlying mechanism in corticosteroid-induced ONFH is still unclear. A previous clinical study reported that the activity of hepatic cytochrome P450 3A, which metabolizes corticosteroids, was related to corticosteroid-induced ONFH [7]. Additionally, several animal studies have reported abnormal hepatic metabolism to be involved in the development of corticosteroid-induced ONFH [8–10]. Therefore, abnormal hepatic metabolism might be associated with the occurrence of corticosteroid-induced ONFH. However, the three case reports on contralateral ONFH did not clearly observe associations with abnormal hepatic metabolism. Little attention has been paid to the associations between development of new corticosteroid-induced ONFH lesions and hepatic abnormality observed in liver biopsy.

Herein, we report the case of a patient who developed new ONFH in the contralateral hip after the dose of corticosteroids for an idiopathic thrombocytopenic purpura (ITP) was increased and who underwent liver biopsy to rule out autoimmune hepatitis.

## Case presentation

**Fig. 1:**
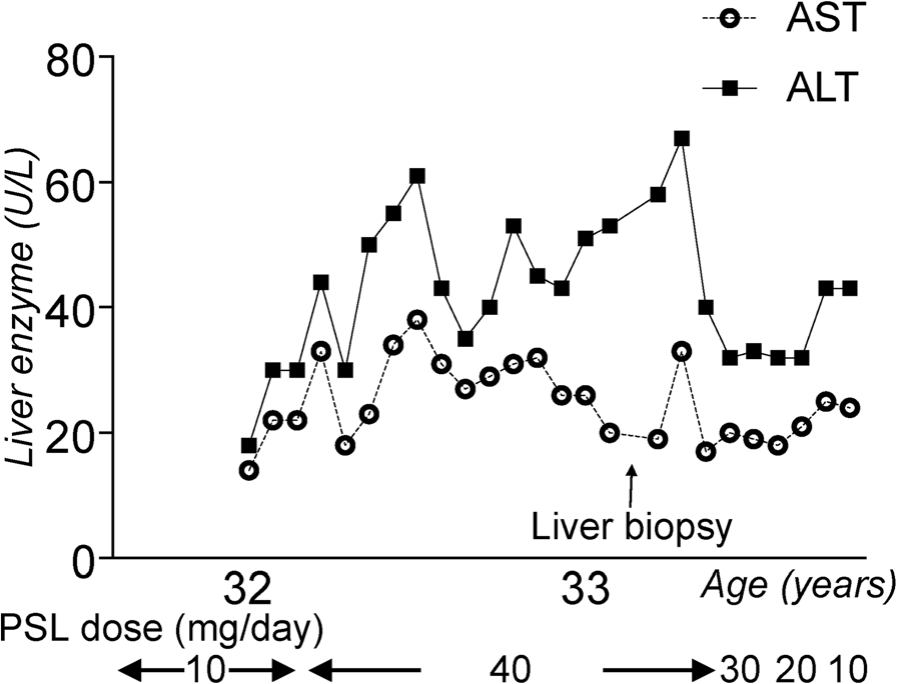
Longitudinal corticosteroid dose and serum liver enzyme level. ○, aspartate aminotransferase (AST); ■, alanine aminotransferase (ALT); PSL, prednisolone

**Fig. 2:**
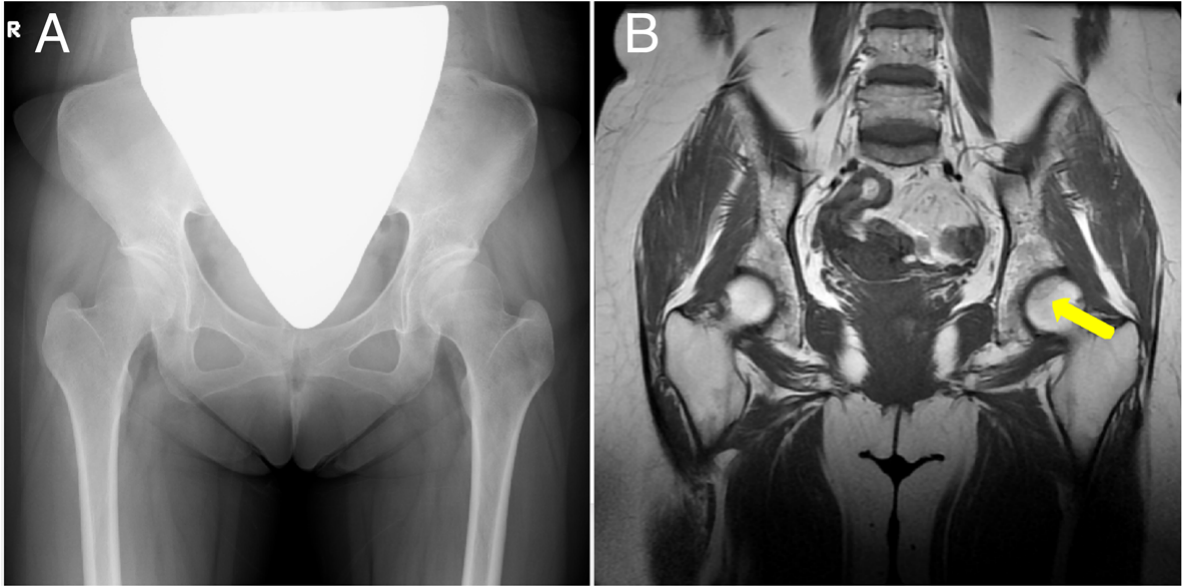
Images obtained at the same month of the onset of hip pain. (**a**) Anteroposterior radiograph of the hip at the first visit. (**b**) Coronal T1-weighted magnetic resonance image of the hip in the same month. Low-intensity band pattern in the left femoral head (arrow)

A 32-year-old woman (height, 150.8 cm; weight, 50.7 kg; body mass index, 22.3 kg/m^2^) had been treated with continuous corticosteroids of up to 10 mg/day for Sjögren’s syndrome since age 8 (Fig. 1). She had no history of alcohol abuse. At age 32, she had groin pain without any antecedent trigger activities. Plain radiography at the same month after onset did not show obvious abnormalities (Fig. 2a). T1-weighted magnetic resonance imaging (MRI) at 3 months after the onset of pain clearly showed a low-intensity band within the left femoral head (Fig. 2b). We diagnosed her with ONFH (type C-2, stage 1) based on the Japanese Investigation Committee (JIC) classification [11, 12]. There was no obvious abnormality in the right femoral head.

**Fig. 3:**
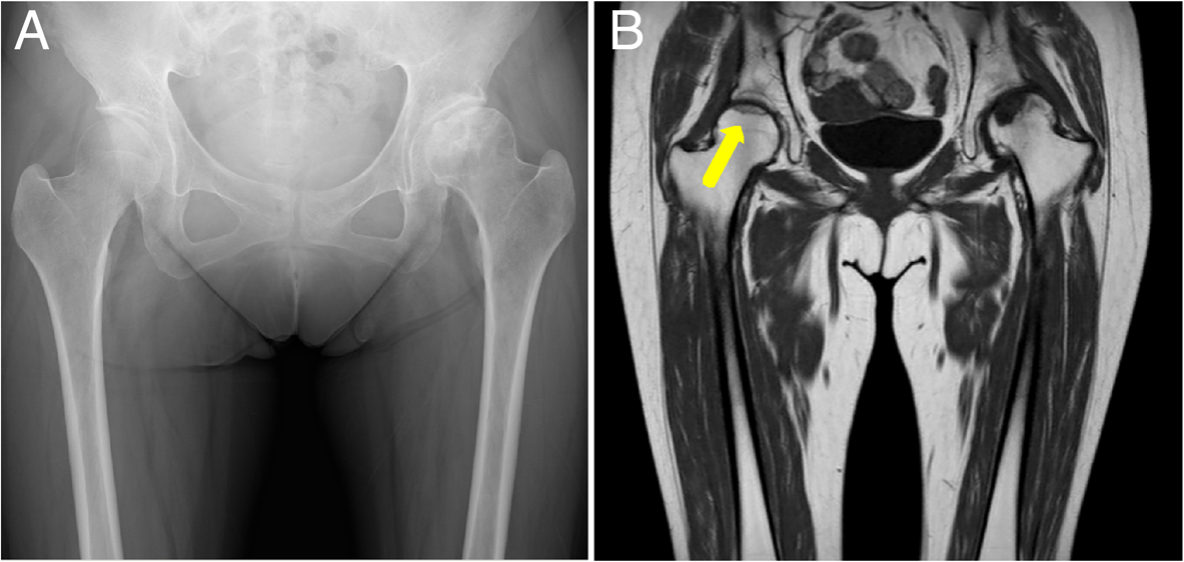
Images obtained at 1 year after corticosteroid dose increase. (**a**) Anteroposterior (AP) radiograph of the hip 1 year after corticosteroid dose increase. (**b**) Coronal T1-weighted magnetic resonance image of the hip in the same month of the AP radiograph. Low-intensity band pattern in the right femoral head (arrow)

At age 33, ITP developed, which was treated by corticosteroid dose increase (40 mg/day) followed by oral corticosteroid therapy (30 mg/day) for 1 year. After the ITP improved, continuous corticosteroid treatment was administered for Sjögren’s syndrome and ITP using a similar regimen as before (10 mg/day). At age 34, 1 year after the corticosteroid dose was increased, T1-weighted MRI demonstrated a low-intensity band within the right femoral head (Fig. 3). Then, she was diagnosed with right ONFH (type C-1, stage 1).

**Fig. 4:**
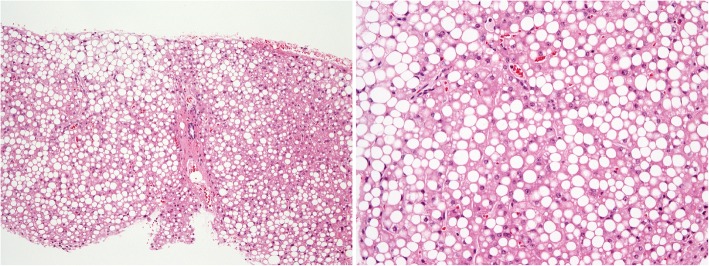
Micrography of the liver biopsy. Left panel: Hematoxylin and eosin (HE) staining of the liver biopsy. Right panel: Higher magnification. HE staining showing hepatocyte steatosis (about 40%). The nonalcoholic steatohepatitis score is 3 (steatosis, 2; lobular inflammation, 0; hepatocyte ballooning, 1). Interface hepatitis is not seen

Serum aspartate aminotransferase and alanine aminotransferase levels were elevated after ITP onset and corticosteroid dose increase, and these levels continued to increase over 1 year (Fig. 1). To rule out autoimmune hepatitis associated with ITP, she underwent a liver biopsy 1 year after the corticosteroid dose was increased. Subsequently, she was diagnosed with nonalcoholic fatty liver disease (NAFLD) induced by corticosteroid treatment (Fig. 4). After the corticosteroid dose was tapered to 10 mg/day, serum liver function improved.

Written informed consent for publication of the case was obtained from the patient.

## Discussion and Conclusions

We present a case of bilateral corticosteroid-induced ONFH in which the timing of development of ONFH in each of the femoral heads and corticosteroid-induced NAFLD differed. On the left side (initial occurrence side), the timing of ONFH occurrence and whether it was corticosteroid-induced ONFH were difficult to determine because a higher dose of glucocorticoid (maximum dose; 10 mg/day) was not administered until the initial occurrence. On the contrary, after the corticosteroid dose was increased from 5 to 10 mg/day to 40 mg/day, ONFH developed on the contralateral side, which did not show evidence of osteonecrosis on the first MRI, indicating the difference in the timing of osteonecrosis development. This finding that increasing corticosteroid dose from 5 to 10 mg/day to 40 mg/day might have induced contralateral ONFH is consistent with a previous report [4]. Koo et al. reported that ONFH occurred in patients who received corticosteroid up to 16 months after initiation of corticosteroid therapy [13], and Piyakunmala et al. reported the high incidence rate and high extension area of asymptomatic osteonecrosis of the contralateral femoral head of the hip in high-risk patients in a cross-sectional study [14]. Therefore, the increase in corticosteroid dose in this case might have reached a new threshold, which triggers ONFH occurrence. Because it was not fully understood whether glucocorticoid dose increase could be a risk factor for contralateral ONFH occurrence, future observational studies are needed.

Consistent with the other three cases of new corticosteroid-induced ONFH development in the contralateral femoral head [4–6], in this case, the occurrence of contralateral ONFH was also seen in a relatively short time after the corticosteroid dose was increased. Additionally, considering the elevation of liver enzymes following an increase in corticosteroid dose as well as the pathological findings of the liver biopsy (corticosteroid-induced NAFLD), abnormal hepatic metabolism induced by corticosteroid treatment might play an important role in ONFH occurrence. However, this is contrary to another clinical report in which a high risk of ONFH in systemic lupus erythematosus (SLE) patients showed no immediate increase in hepatic enzyme under steroid therapy [10]. Okazaki et al. reported that the absence of any response to steroid therapy in the liver may be implicated in the pathogenesis of ONFH [10]. However, the current case is different from Okazaki’s study in terms of the duration of corticosteroid intake and the lack of SLE.

Several animal studies focused on the correlation between cytochrome P450 3A activity and the incidence of osteonecrosis [8] [15]. Additionally, clinical reports showed that low hepatic cytochrome P450 3A activity is a risk factor for corticosteroid-induced osteonecrosis [7] [16]. Unfortunately, our patient was not examined then. Therefore, whether abnormal hepatic metabolism could directly play an important role in ONFH occurrence remains unclear. However, recent reports showed the association between reduced level of cytochrome P450 3A activity and expression in NAFLD [17] as well as between corticosteroid use and NAFLD [18]. Given this finding, abnormal hepatic metabolism and hepatic steatosis after corticosteroid dose increase or high-dose corticosteroid initiation should be monitored. Additionally, future observational studies on corticosteroid dose increase should focus on cytochrome P450 3A activity and address the correlation of ONFH occurrence and liver enzyme abnormalities.

The current case indicates that corticosteroid dose increase is a potential risk factor for NAFLD and contralateral ONFH. Therefore, it would be useful and important to screen and monitor patients with hepatic enzyme abnormality for ONFH occurrence.

## Abbreviations

ITP: idiopathic thrombocytopenic purpura
NAFLD: nonalcoholic fatty liver disease
ONFH: osteonecrosis of the femoral head
SLE: systemic lupus erythematosus
